# A Mitochondrial-targeted purine-based HSP90 antagonist for leukemia therapy

**DOI:** 10.18632/oncotarget.23097

**Published:** 2017-12-11

**Authors:** Kelly G. Bryant, Young Chan Chae, Rogelio L. Martinez, John C. Gordon, Khaled M. Elokely, Andrew V. Kossenkov, Steven Grant, Wayne E. Childers, Magid Abou-Gharbia, Dario C. Altieri

**Affiliations:** ^1^ Prostate Cancer Discovery and Development Program, The Wistar Institute, Philadelphia, PA, USA; ^2^ Immunology, Microenvironment and Metastasis Program, The Wistar Institute, Philadelphia, PA, USA; ^3^ Moulder Center for Drug Discovery Research, School of Pharmacy, Temple University, Philadelphia, PA, USA; ^4^ Department of Pharmaceutical Chemistry, Tanta University, Tanta, Egypt; ^5^ Center for System and Computational Biology, The Wistar Institute, Philadelphia, PA, USA; ^6^ Department of Medicine and Massey Cancer Center, Virginia Commonwealth University, Richmond, VA, USA

**Keywords:** mitochondria, chaperone, Hsp90, acute myeloid leukemia, metabolism

## Abstract

Reprogramming of mitochondrial functions sustains tumor growth and may provide therapeutic opportunities. Here, we targeted the protein folding environment in mitochondria by coupling a purine-based inhibitor of the molecular chaperone Heat Shock Protein-90 (Hsp90), PU-H71 to the mitochondrial-targeting moiety, triphenylphosphonium (TPP). Binding of PU-H71-TPP to ADP-Hsp90, Hsp90 co-chaperone complex or mitochondrial Hsp90 homolog, TRAP1 involved hydrogen bonds, π-π stacking, cation-π contacts and hydrophobic interactions with the surrounding amino acids in the active site. PU-H71-TPP selectively accumulated in mitochondria of tumor cells (17-fold increase in mitochondria/cytosol ratio), whereas unmodified PU-H71 showed minimal mitochondrial localization. Treatment of tumor cells with PU-H71-TPP dissipated mitochondrial membrane potential, inhibited oxidative phosphorylation in sensitive cell types, and reduced ATP production, resulting in apoptosis and tumor cell killing. Unmodified PU-H71 had no effect. Bioinformatics analysis identified a “mitochondrial Hsp90” signature in Acute Myeloid Leukemia (AML), which correlates with worse disease outcome. Accordingly, inhibition of mitochondrial Hsp90s killed primary and cultured AML cells, with minimal effects on normal peripheral blood mononuclear cells. These data demonstrate that directing Hsp90 inhibitors with different chemical scaffolds to mitochondria is feasible and confers improved anticancer activity. A potential “addiction” to mitochondrial Hsp90s may provide a new therapeutic target in AML.

## INTRODUCTION

Despite the pervasive glycolytic metabolism of most tumors, the so-called “Warburg effect” [1], there is now resurgent interest in the role of mitochondria in cancer [2, 3]. This is because mitochondrial bioenergetics is preserved in most malignancies [4], ATP produced via oxidative phosphorylation supports tumor growth, *in vivo* [5], and reprogramming of mitochondrial functions promotes key cancer traits, including drug resistance [6], “stemness” [7], and disease dissemination to distant organs, or metastasis [8, 9].

Against this backdrop, drug discovery efforts have focused on targeting mitochondrial functions for cancer therapy [10]. Although modulation of Bcl2-dependent apoptosis at the outer mitochondrial membrane is feasible [11], and has entered clinical practice [12], therapeutic manipulation of mitochondrial mechanisms of bioenergetics, ROS production, and protein and nucleic acid metabolism is still in infancy [13]. Recent evidence suggests that these pathways rely on heightened protein folding quality control mediated by mitochondria-localized chaperones of the Heat Shock Protein-90 (Hsp90) family [14], including Hsp90 and its homolog, TNFR-Associated Protein-1 (TRAP1). Accordingly, these molecules prominently accumulate in mitochondria of most tumors, compared to normal cells [14], where they buffer proteotoxic stress [15, 16], maintain a multifunctional mitochondrial “proteome” [17], and sustain primary and metastatic tumor growth, *in vivo* [18, 19].

Molecular chaperones, and Hsp90 in particular, are recognized as important cancer drivers [20], and actionable therapeutic targets [21]. However, the role of the mitochondria-localized Hsp90s is controversial, variously linked to tumor promotion or suppression, and activation or inhibition of oxidative metabolism [22]. To dissect these pathways, a well-characterized benzoquinone ansamycin Hsp90 inhibitor, 17-allylaminogeldanamycin (17-AAG) [21] was made mitochondria-permeable. Designated as Gamitrinib (GA mitochondrial matrix inhibitor) [23], this compound selectively accumulated in mitochondria [23], triggered an organelle unfolded protein response [15, 16], and delivered superior anticancer activity, compared to unmodified 17-AAG [13]. However, the specificity of these responses has not been clearly established, and the potential sensitivity of hematopoietic malignancies to this potential therapeutic approach has not been clearly demonstrated.

In this study, we synthesized and characterized two mitochondria-targeted Hsp90 inhibitors with a purine-based chemical scaffold derived from PU-H71 [24].

## RESULTS

### Chemical synthesis of mitochondria-targeted, Hsp90 inhibitors H71-TPP-1 and H71-TPP-2

**Figure 1:**
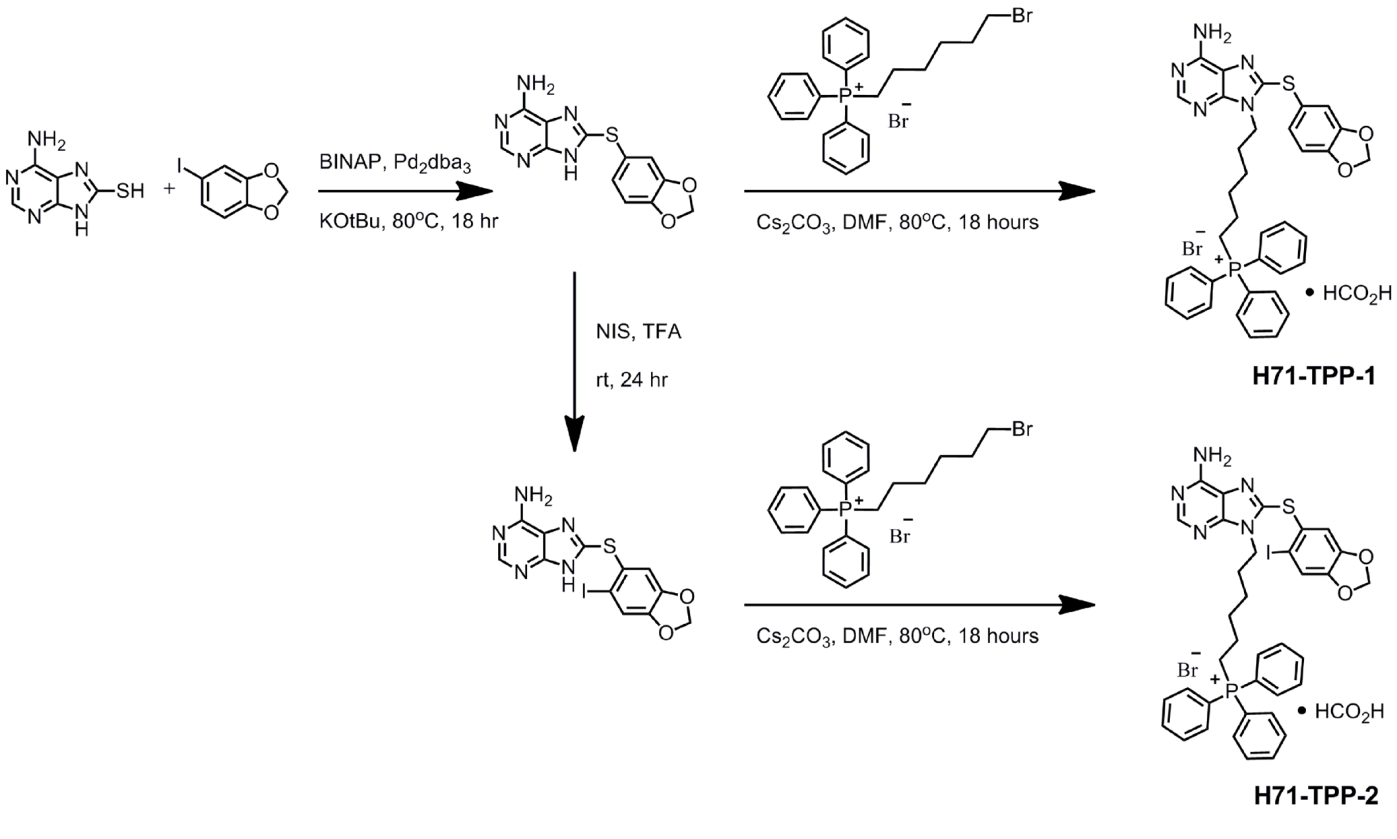
Chemical synthesis of mitochondrial-targeted small molecule Hsp90 inhibitor PU-H71-TPP The individual synthesis steps and corresponding experimental conditions are indicated. The two final compounds used in this study H71-TPP-1 and H71 TPP-2 differ from the absence or presence of a iodo substituent on the methylenedioxy moiety, respectively.

The chemical synthesis of two mitochondrial-targeted versions of PU-H71 [24] is shown in Figure 1. Similar to Gamitrinib [23], PU-H71 and its desi-iodo analog were made mitochondria-targeted by the addition of triphenylphosphonium (TPP) (Figure 1). Two variants of PU-H71-TPP were synthesized, depending on the absence (H71-TPP-1) or presence (H71-TPP-2) of an iodo substituent on the methylenedioxy moiety. The des-iodo analog H71-TPP-1 was used for quantification of subcellular fractions. The more potent iodinated derivative H71-TPP-2 was used for most of the follow-up studies, unless otherwise specified.

### Molecular modeling of mitochondria-targeted H71-TPP-2 ligand binding

**Figure 2:**
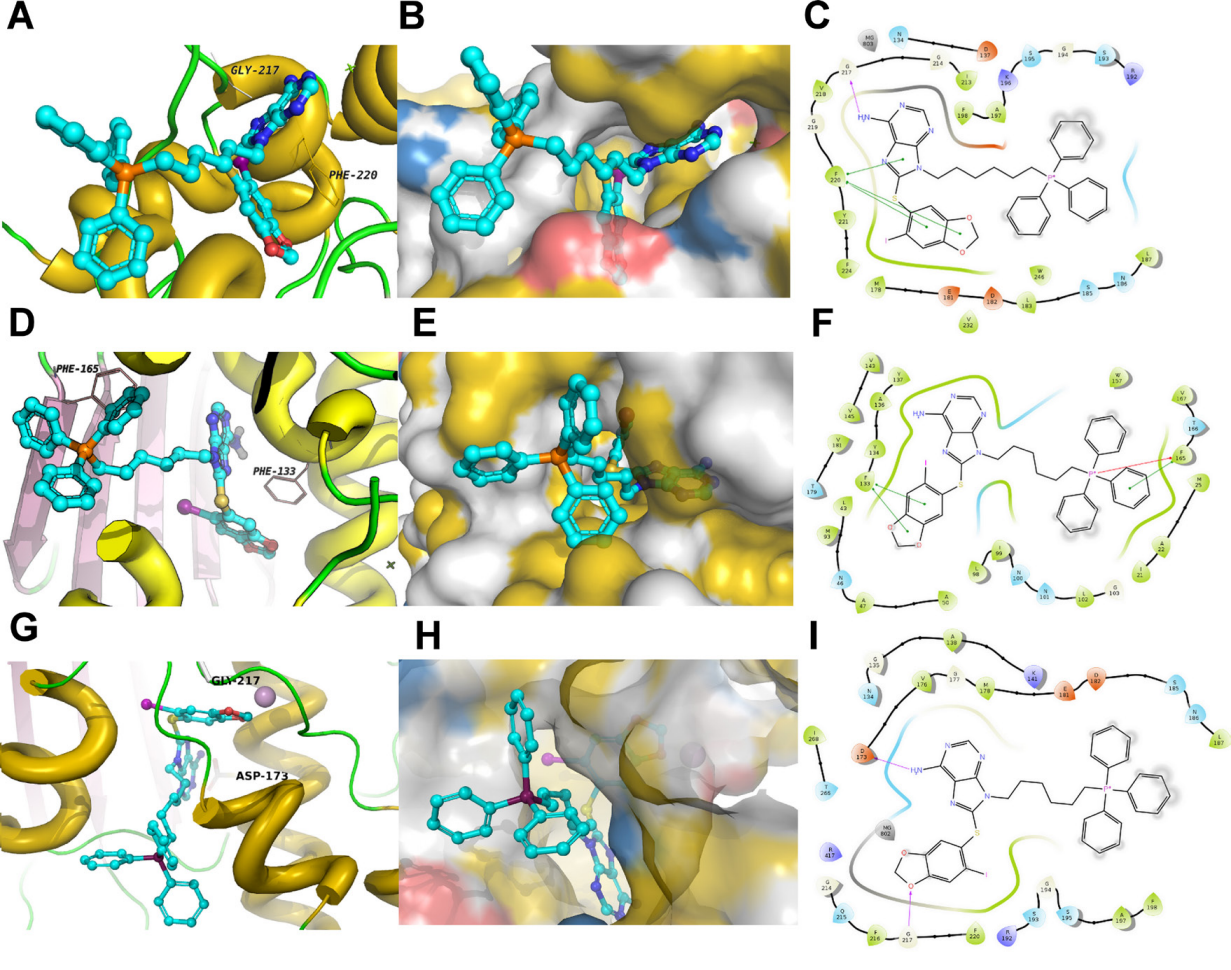
Predicted model of PU-H71-TPP (H71-TPP-2) target binding **A**, **B** and **C**. ADP-Hsp90 complex. **D**, **E** and **F**. Co-chaperone-Hsp90 complex. **G**, **H** and **I**. TRAP1. For each condition, the 3D interaction model of the ligand in the binding site showing the interacting amino acids as lines (**A, D** and **G**), and the binding mode of the ligand with hydrophobic surface representation colored by YRB highlighting scheme (**B, E** and **H**) is shown. **C, F** and **I**. 2D interaction diagram. For panels **B, E** and **H**, yellow is used for non-polar hydrocarbons; red for negatively charged oxygens of glutamate and aspartate; blue for positively charged nitrogens of lysine and arginine; blue and white for all remaining atoms including the polar backbone.

**Figure 3:**
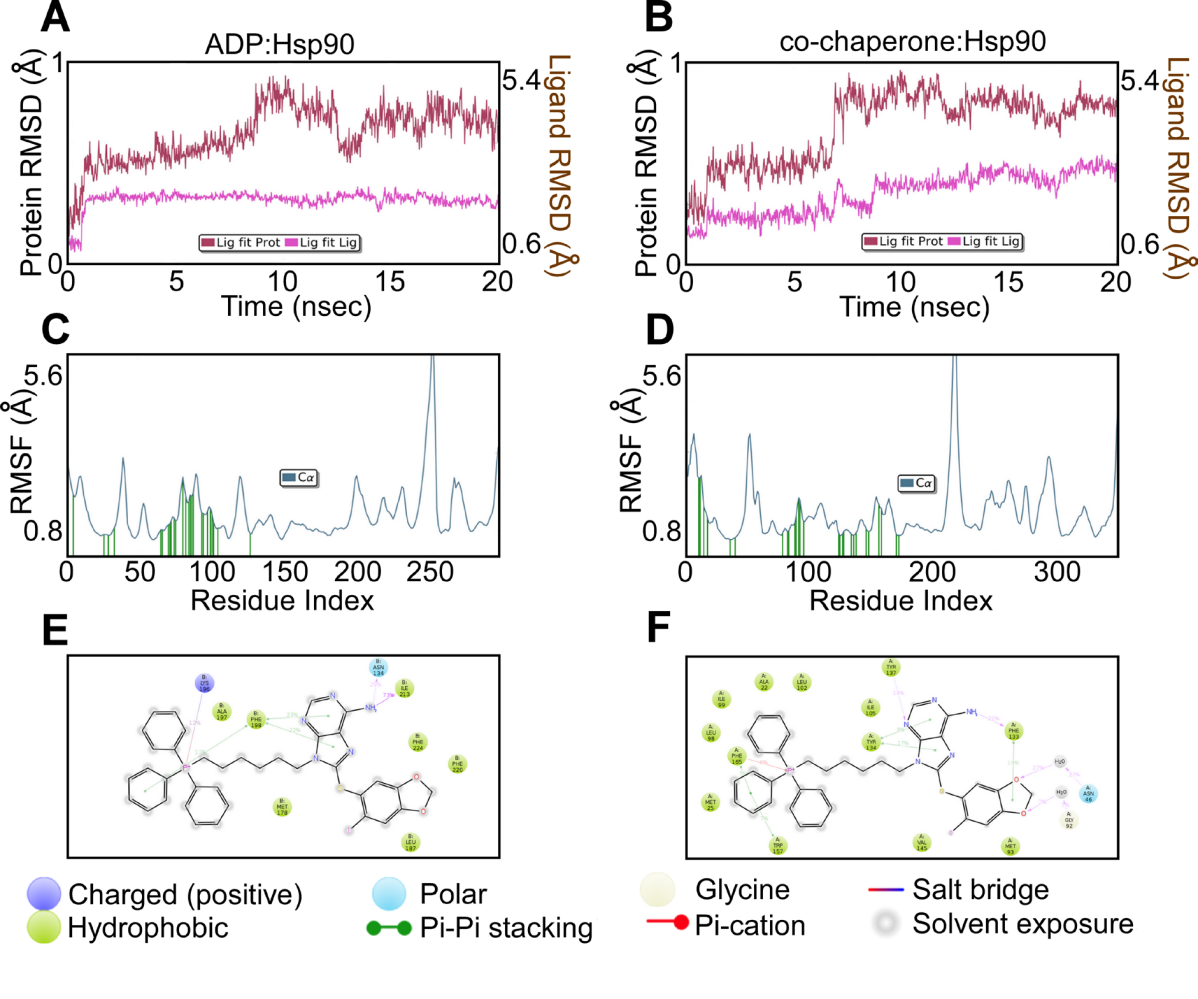
Ligand binding parameters **A** and **B**. Ligand RSMD of PU-H71-TPP (H71-TPP-2) binding to ADP-Hsp90 or co-chaperone-Hsp90 complex. The average change in displacement of ligand atoms is shown with respect to the initial binding mode during the course of MD simulation. ‘Lig fit Prot’ shows the RMSD of a ligand when the protein-ligand complex is compared to the backbone of the initial binding mode complex. ‘Lig fit Lig’ shows the RMSD of the ligand when compared to its initial conformation. **C** and **D**. Ligand RSMF of PU-H71-TPP (H71-TPP-2) predicted binding to ADP-Hsp90 or co-chaperone-Hsp90 complex. Ligand contacts are the protein residues that interact with the ligand and are shown as green vertical bars. **E** and **F**. Schematic representation of the protein-ligand interactions that occur >10% of simulation time for binding to ADP-Hsp90 or co-chaperone-Hsp90 complex.

To rule out non-specific effects due to the addition of TPP, the binding mode of H71-TPP-2 to Hsp90 bound to ADP (PDB ID: 2IOP), an Hsp90 co-chaperone complex involving Cdc37 and Cdk4 (PDB ID: 5FWP), as well as TRAP1 (PDB ID: 4IPE), was evaluated (Figure 2). In these studies, H71-TPP-2 docked in ADP:Hsp90, the co-chaperone:Hsp90 complex and TRAP1 with docking scores of -3.6 kcal/mol, -7.8 kcal/mol, and -3.4 kcal/mole, respectively. In all three proteins, the TPP moiety was solvent-exposed. H71-TPP-2 binding to Hsp90:ADP and to the co-chaperone:Hsp90 complex was aided by π-π and cation-π contacts with nearby amino acid residues, interactions that were not observed when H71-TPP-2 was docked to TRAP1. According to this model, H71-TPP-2 forms two hydrogen bonds with Gly217 and Lys196, π-π contact with Phe220, and several interactions with the surrounding amino acids of the ADP:Hsp90 complex (Figure 2A, and 2C). The ligand forms similar contacts with the co-chaperone:Hsp90 complex, including π-π contact with Phe133, and π-π/cation-π contacts with Phe165 (Figure 2D, and 2F). H71-TPP-2 formed hydrogen bonds with Asp173 and Gly217 as well as a strong ion-dipole interaction with the Mg^+2^ ion of TRAP1, but did not form any interactions involving the TPP group (Figure 2G, and 2I). The calculated Prime MM-GBSA binding energy of the ligand was -40 kcal/mol for the ADP:Hsp90 complex and -90 kcal/mol for the co-chaperone:Hsp90 complex, consistent with high ligand-binding affinities. Prime MM-GBSA binding energy of the ligand was 10-fold less (-3.4 kcal/mol) for TRAP1, suggesting a lower binding affinity. The constancy of the two high affinity binding modes was further investigated by running 20 ns molecular dynamics (MD) simulation. The ligand RMSD was calculated over the course of the simulation time to evaluate the stability of the docking pose (Figure 3A and 3B). The internal fluctuations of the ligand atoms demonstrated high stability, as shown by the ‘Lig fit Prot’ and the ‘Lig fit Lig’ plots (Figure 3A and 3B).

The local changes along protein chains were studied for the two high affinity binding modes by calculating the root mean square fluctuation (RMSF, Figure 3C and 3D). H71-TPP-2 contacts were observed in the area with the least protein fluctuation, consistent with the steadiness of the ligand in the binding pocket. The same protein-ligand contacts observed from the docking simulations were also detected during MD simulations (Figure 3E and 3F) in addition to other strong interactions, including hydrogen bonding with Asn134 and Ile213, π-π contacts with Phe198, and cation-π interactions with Lys196 in case of the ADP:Hsp90 complex. Additional interactions were observed for ligand binding in the co-chaperone:Hsp90 complex, including hydrogen bonding with Tyr137 and Phe133, interactions through water bridges with Asn46 and Gly92, and π-π contacts with Tyr134 and Trp157. In all binding models, including TRAP1, the TPP moiety showed high solvent exposure. However, whereas π-π and cation-π interactions stabilized the binding of the TPP group with the surface amino acid residues of Hsp90:ADP and co-chaperone:Hsp90 complex, the lack of such interactions destabilized TPP binding to TRAP1, contributing to the predicted 10-fold lower binding energy. By contrast, docking simulation studies indicated that Gamitrinib bound more tightly to TRAP1, compared to H71-TPP-2. In these experiments, the Gamitrinib binding mode involved several hydrogen bonds with amino acid residues in the ligand pocket, including Gln215, Asn134, Phe220, Asp173, Thr266, Lys141 and Ser195. The linker shows hydrogen bonds with Ser195 and Ile213, whereas the flexibility of the macrocyclic ring of Gamitrinib inside the binding pocket allows for better fitting.

### Mitochondrial targeting of H71-TPP

**Figure 4:**
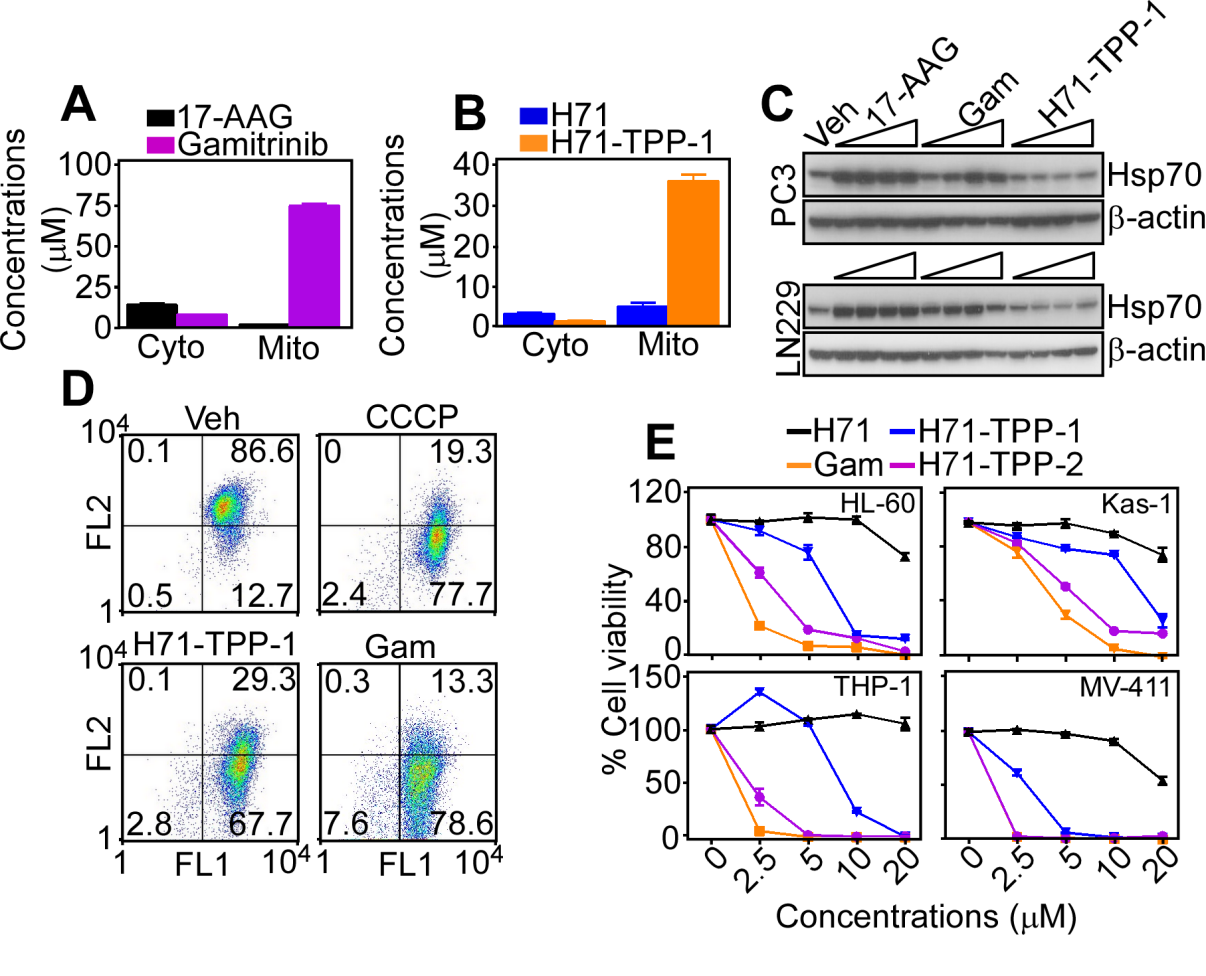
Mitochondria-targeted PU-H71 causes acute organelle dysfunction **A**. LC/MS quantification of Gamitrinib or 17-AAG or **B**. PU-H71 (H71) or H71-TPP-1 (H71-TPP) in isolated subcellular fractions of treated PC3 cells. Cyto, cytosol; Mito, mitochondria; M/C, mitochondria/cytosol ratio. Data are the mean±SD (n=2). **C**. The indicated tumor cell lines were treated with vehicle (Veh), TPP alone, or increasing concentrations (2.5, 5, 10, 20 μM) of 17-AAG, Gamitrinib or H71-TPP-1 (H71-TPP) and analyzed by Western blotting. **D**. AML HL-60 cells were incubated with vehicle (Veh) or H71-TPP-1 (H71-TPP) (2.5, 5 μM), and analyzed for mitochondrial membrane potential by JC1 staining and multiparametric flow cytometry. The percentage of cells in each quadrant is indicated. The mitochondrial uncoupler CCCP was used as control. FL, fluorescence. **E**. The indicated AML cell lines were incubated with increasing concentrations of Gamitrinib (Gam), PU-H71 (H71), PU-H71-TPP-1 or PU-H71-TPP-2 and analyzed for cell viability by an alamarBlue assay. Data are the mean±SD (n=3).

LC/MS analysis of subcellular fractions from treated PC3 cells demonstrated that Gamitrinib selectively accumulated in mitochondria, with a 106-fold enrichment in mitochondria compared to cytosol (Figure 4A), in agreement with previous observations [23]. Unmodified 17-AAG was undetectable in mitochondria (Figure 4A). In these experiments, H71-TPP-1 (H71-TPP) also accumulated in tumor mitochondria, with a 17-fold enrichment in mitochondria, compared to cytosol (Figure 4B). Unmodified PU-H71 (H71) minimally localized to mitochondria (Figure 4B). Consistent with selective mitochondrial localization, H71-TPP-1 did not increase Hsp70 levels in tumor cells, a marker of Hsp90 inhibition in cytosol, whereas Gamitrinib had minimal effect (Figure 4C). Functionally, treatment of AML HL-60 cells with H71-TPP-1 resulted in concentration-dependent loss of mitochondrial membrane potential, by JC1 staining and multiparametric flow cytometry (Figure 4D). Gamitrinib also dissipated mitochondrial membrane potential in HL60 cells, more potently than H71-TPP-1 (Figure 4D). Consistent with mitochondrial defects, increasing concentrations of H71-TPP-1, H-71-TPP-2 or Gamitrinib reduced the viability of a panel of AML cell lines (Figure 4E). In these experiments, H71-TPP-2 was more effective than H71-TPP-1 and comparable to Gamitrinib for anti-AML activity, whereas under the same experimental conditions, unconjugated H71 was ineffective (Figure 4E).

### Inhibition of mitochondrial Hsp90s induces metabolic defects and apoptotic cell death

**Figure 5:**
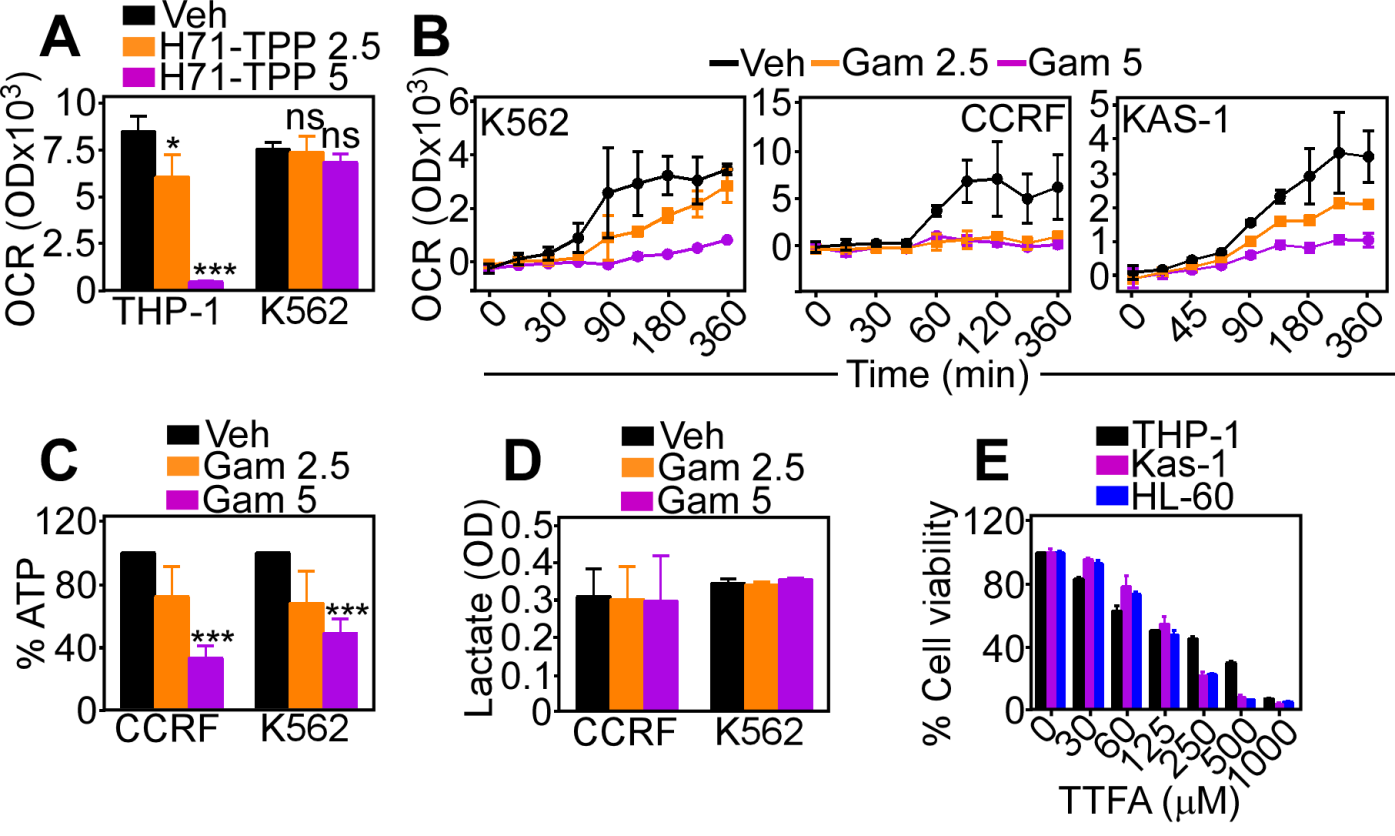
Inhibition of mitochondrial Hsp90 induces metabolic defects **A**. The indicated AML cell lines were incubated with vehicle (Veh) or H71-TPP-2 (H71-TPP) (2.5-5 μM) and analyzed for oxygen consumption rates (OCR) after a 360-min incubation. Data are the mean±SD (n=3). ^*^, p=0.04; ^***^, p<0.0001; ns, not significant. **B**. The indicated AML cell types were incubated with Gamitrinib (2.5-5 μM) and analyzed for OCR at increasing time intervals. Data are the mean±SD (n=3). **C**. The indicated AML cell types were incubated with Gamitrinib (Gam, 2.5-5 μM) and analyzed for ATP production or **D**. lactate generation. Data are the mean±SD (n=3). ^***^, p= 0.0006-<0.0001. **E**. The indicated AML cell types were incubated with increasing concentrations of mitochondrial oxidative phosphorylation Complex II inhibitor, TTFA and analyzed for cell viability by an alamarBlue assay. Data are the mean±SD (n=3).

Treatment with H71-TPP-2 (H71-TPP) inhibited oxygen consumption rates (OCR) in THP-1 cells, whereas a CML cell type, K562 was largely resistant (Figure 5A). By comparison, Gamitrinib was a more potent inhibitor of oxidative bioenergetics than H71-TPP, and indistinguishably suppressed OCR in AML or CML cell types in a concentration-dependent manner (Figure 5B). This was associated with dose-dependent inhibition of ATP production (Figure 5C), whereas lactate generation, a marker of glycolysis, was not affected (Figure 5D). Finally, treatment with TTFA, a small molecule inhibitor of mitochondrial Complex II, killed AML cell types in a dose-dependent manner (Figure 5E), suggesting that oxidative metabolism was required for AML maintenance.

**Figure 6:**
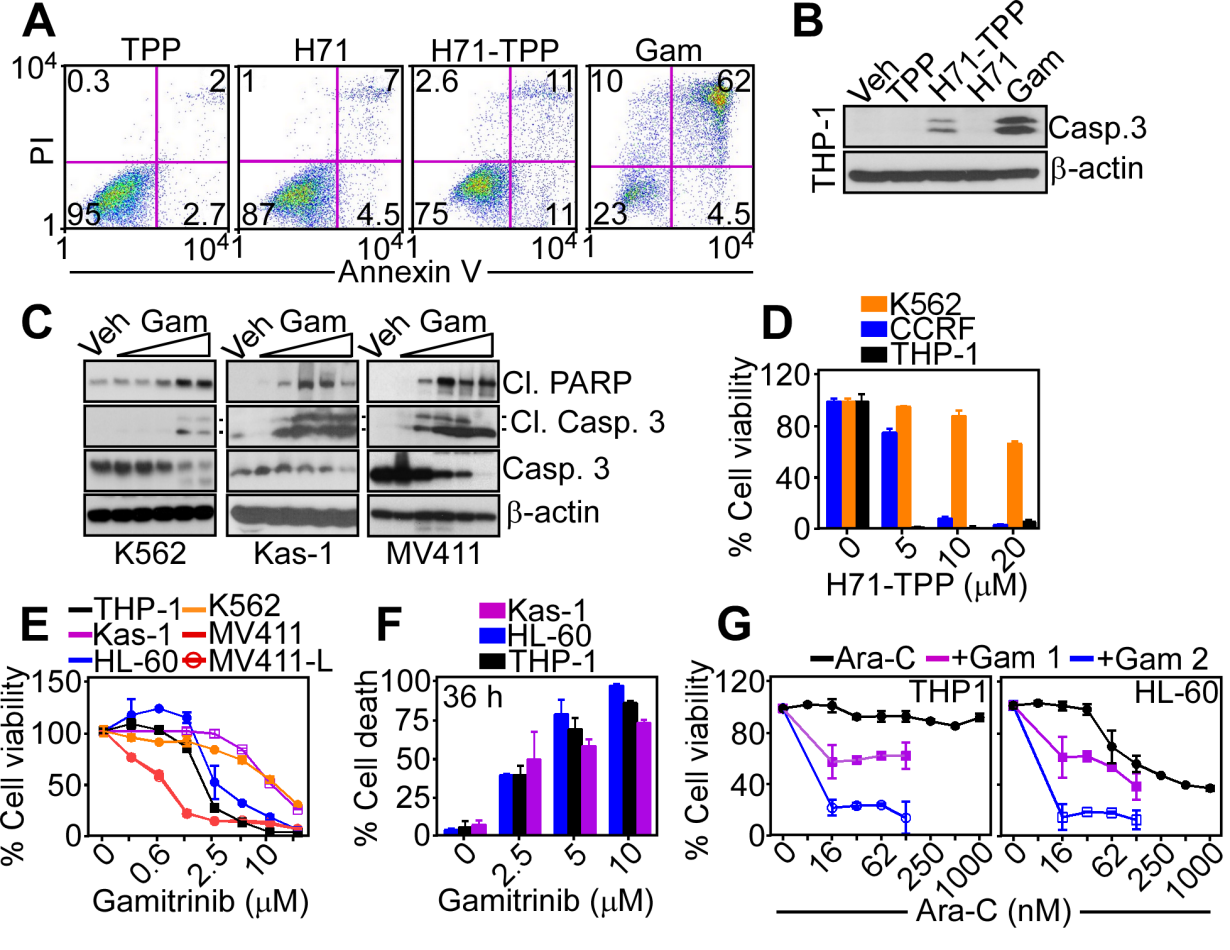
PU-H71-TPP induces tumor cell death **A**. PC3 cells were treated with vehicle (Veh) or the indicated agents (all at 20 μM, H71-TPP-2 (H71-TPP)) and analyzed for Annexin V and propidium iodide (PI) staining by multiparametric flow cytometry. The percentage of cells in each quadrant is indicated. **B**. THP-1 cells were treated with the indicated agents and analyzed by Western blot for caspase cleavage. **C**. The indicated leukemia cell lines were treated with increasing concentrations of Gamitrinib (Gam, 2.5, 5, 10 μM) and analyzed by Western blotting. The positions of cleaved PARP or cleaved caspase-3 subunits are indicated. **D**. The indicated AML cell types were incubated with increasing concentrations of H71-TPP-2 (H71-TPP) and analyzed for cell viability by an alamarBlue assay. Data are the mean±SD (n=3). **E**. AML cell lines were incubated with increasing concentrations of Gamitrinib and analyzed for cell viability by an alamarBlue assay. Data are the mean±SD (n=3). **F**. The experimental conditions are as in E. except that the indicated treated cultures were quantified for cell death by direct cell counting after 36 h. Data are the mean±SD (n=4). **G**. The indicated AML cell lines were incubated with increasing concentrations of Ara-C, alone or in combination with 1 μM or 2 μM Gamitrinib (Gam) and analyzed for cell viability by an alamarBlue assay. Data are the mean±SD (n=2).

In addition to inhibition of bioenergetics, exposure of tumor cells to Gamitrinib or H71-TPP-2 (H71-TPP) triggered apoptosis, characterized by increased Annexin V reactivity, by multiparametric flow cytometry (Figure 6A), and cleavage of effector caspase 3 (Figure 6B), by Western blotting. Confirming the specificity of this response, the TPP moiety alone or unconjugated H71 had no effect on Annexin V reactivity (Figure 6A) or active caspase-3 generation (Figure 6B). Similar results were obtained with Gamitrinib treatment of a panel of AML cell types, which also resulted in hallmarks of apoptosis, including PARP and caspase-3 cleavage (Figure 6C). Consistent with these data, H71-TPP induced concentration-dependent killing of leukemia cell lines, CCRF-CEM and THP-1, whereas K562 cells were resistant (Figure 6D), potentially reflecting a cytoprotective role of BCR-ABL signaling under these conditions. Conversely, Gamitrinib was effective on all leukemia cell lines tested (Figure 6E), achieving complete cell killing within 36 h of drug exposure (Figure 6F). In addition, exposure of AML THP-1 or HL-60 cells to cytosine arabinoside (Ara-C), which is standard of care for leukemia treatment, did not significantly reduce cell viability (Figure 6G). However, the addition of low, minimally cytotoxic concentrations of Gamitrinib enhanced the anti-leukemic activity of Ara-C against AML cell types in a concentration-dependent manner (Figure 6G).

### Mitochondrial Hsp90 expression in AML

**Figure 7:**
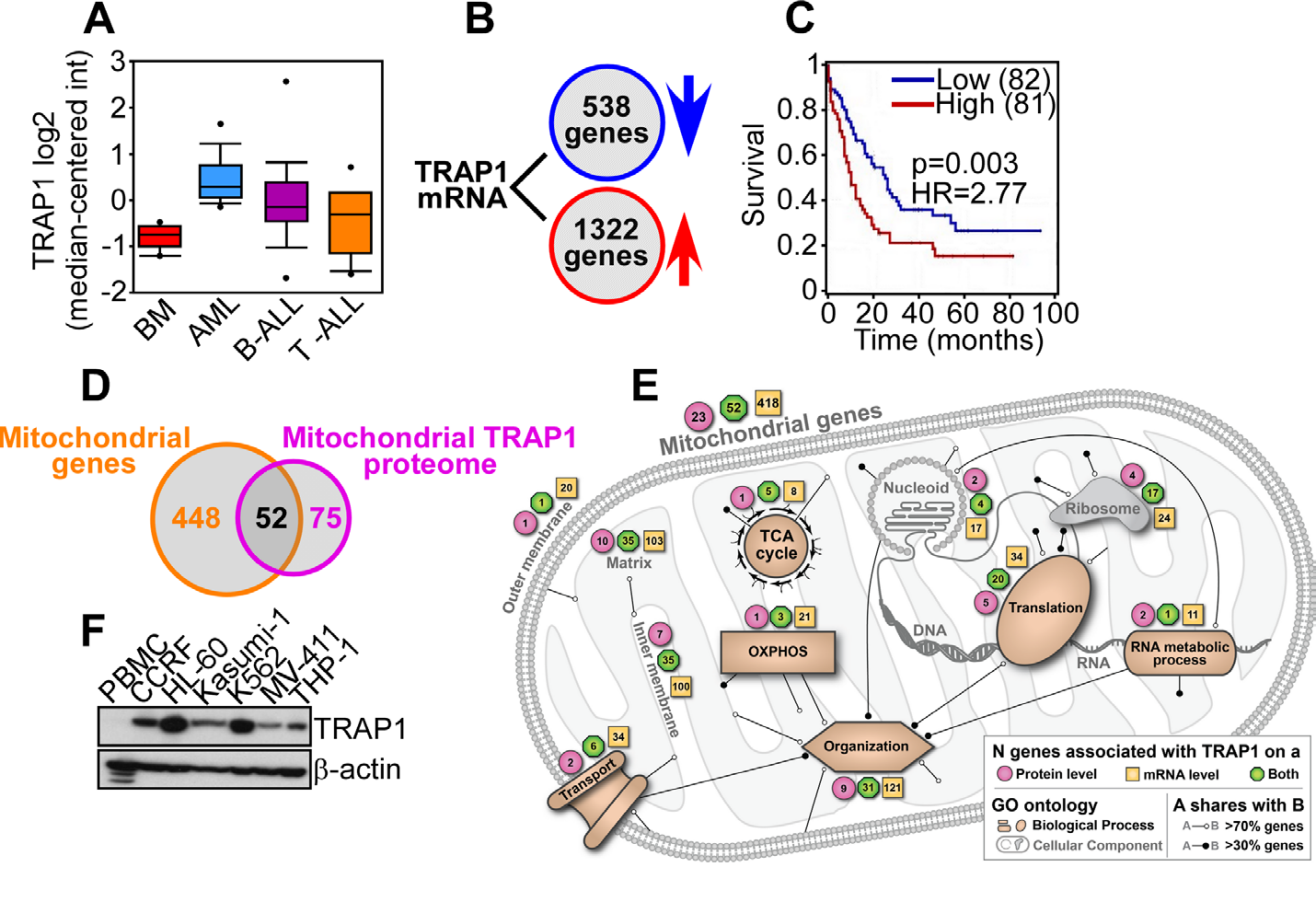
Bioinformatics analysis of TRAP1 expression in AML **A**. Oncomine analysis of TRAP1 mRNA expression in normal bone marrow (n=6), AML (n=23), B-cell ALL (n=87) or T-cell ALL (n=11) [40]. TRAP1 mRNA expression in normal bone marrow *versus* AML: 2.39-fold induction, p=7.98×10^-8^. **B**. Identification of 1,322 genes positively correlated with TRAP1 expression and 538 genes negatively correlated with TRAP1 expression (FDR<5%, Spearman |r|>0.3) in AML TCGA mRNA-seq data. **C**. Kaplan-Meier curve of differential AML survival based on the combined mRNA profile of genes associated with high or low TRAP1 expression (p=0.003, HR=2.77 by Cox regression). **D**. Genes positively correlated with TRAP1 expression are enriched in mitochondria-related genes (2.2-fold enrichment, p<10^-10^ by hypergeometric test), and comprise 70% (52 proteins) of mitochondrial proteins that require TRAP1 for proper folding (4.9-fold enrichment, p<10^-10^ by hypergeometric test). **E**. Combined schematic model of mitochondrial functions and cellular pathways associated with the TRAP1 protein and gene signature in AML. **F**. Peripheral blood normal mononuclear cells (PBMC) or leukemia cell lines were analyzed by Western blotting.

Bioinformatics analysis of public databases revealed that TRAP1 was prominently upregulated in pediatric AML patients, compared to normal bone marrow, or B- or T-cell Acute Lymphoblastic Leukemia (ALL) (Figure 7A). Elevated TRAP1 levels were also observed in adult AML, irrespective of FAB (French-American-British) subtype (M1-M6) or genetic alterations, including 11q23MLL gene rearrangement, CBFB-MYH1 gene fusion, FLT3 mutation, PML-RARα translocation, and RUNX1-RUNX1T1 translocation (not shown). Examination of TCGA mRNA-seq data identified 1322 genes positively correlated with TRAP1 expression, and 538 genes (FDR<5%, Spearman |r|>0.3) negatively correlated with TRAP1 expression (Figure 7B). Using a Cox regression model with age and gender as co-variates, the combined mRNA profile of the genes that corresponded to higher TRAP1 expression correlated with shortened overall survival in AML (p=0.003, hazard ratio HR=2.77) (Figure 7C). In addition, genes positively correlated with TRAP1 were significantly enriched in mitochondria-related genes (2.2-fold enrichment, p<10^-10^ by hypergeometric test), and 52 of the 75 mitochondrial proteins that require TRAP1 for proper folding (70%) [17] showed mRNA association with TRAP1: an overlap of 4.9-fold over a randomly expected association (p<10^-10^ by hypergeometric test) (Figure 7D). By pathway analysis, these proteins contributed to multiple mitochondrial functions, including oxidative phosphorylation, transport, and protein and nucleic acid processing (Figure 7E). Consistent with these findings, TRAP1 was highly expressed in various AML cell types, whereas normal peripheral blood mononuclear cells (PBMC) were unreactive (Figure 7F).

**Figure 8:**
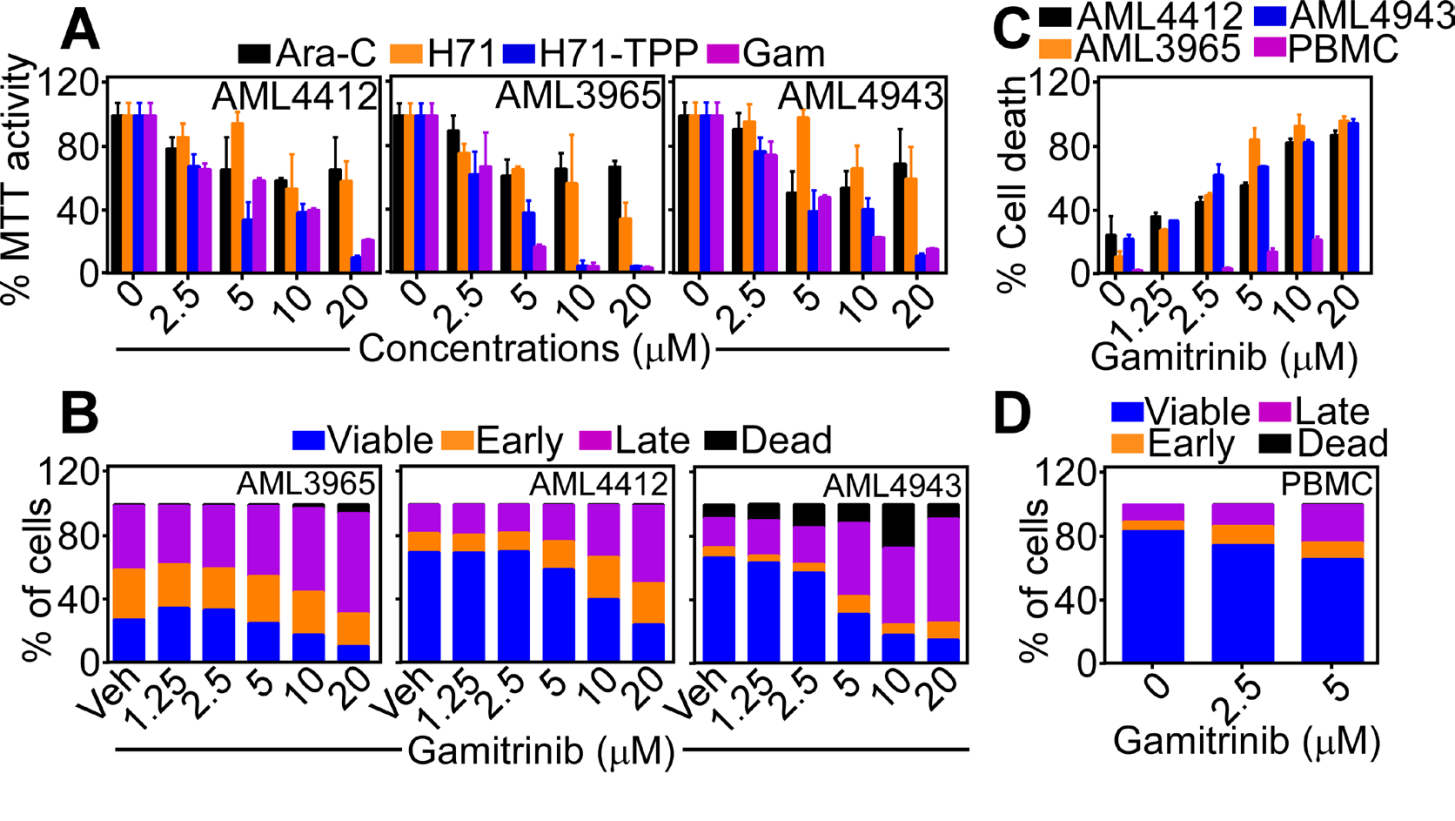
Inhibition of mitochondrial Hsp90s for AML therapy **A**. The indicated patient-derived AML samples were treated with increasing concentrations of Gamitrinib or H71-TPP-2 (H71-TPP) and analyzed for mitochondrial function by an MTT assay. Data are the mean±SD of replicates of a representative experiment. **B**. The experimental conditions are as in A. except that treated AML samples were analyzed by Annexin V/PI staining and multiparametric flow cytometry. The percentage of viable cells or cells in different apoptotic stages (early, late, dead) was quantified. **C**. Gamitrinib-treated primary AML samples or PBMC were analyzed for cell death by direct cell counting. Data are the mean±SD of replicates of a representative experiment. **D**. Normal human PBMC were treated with the indicated concentrations of Gamitrinib and analyzed for Annexin V/PI staining and flow cytometry, with quantification of viable cells or cells in the various apoptotic phases (early, late, dead).

Functionally, treatment of primary, patient-derived AML samples with Gamitrinib or H71-TPP-2 (H71-TPP) induced concentration-dependent loss of mitochondrial function, by an MTT assay (Figure 8A). This was associated with potent induction of mitochondrial apoptosis in primary AML samples, by Annexin V staining and multiparametric flow cytometry (Figure 8B), which culminated in nearly complete cell killing within 36 h of drug exposure (Figure 8C). Under the same conditions, Gamitrinib did not significantly affect Annexin V reactivity (Figure 8D), or viability of normal PBMC (Figure 8C).

## DISCUSSION

In this study, we have shown that subcellular targeting of small molecule Hsp90 antagonists to mitochondria is feasible, “portable” across diverse chemical scaffolds, and confers much improved anticancer activity compared to the corresponding, non-targeted compounds. In the case of the purine-based Hsp90 antagonist investigated here, PU-H71 [24, 25], mitochondrial targeting provided by a TPP moiety [23] triggered organelle dysfunction, inhibition of oxidative metabolism in sensitive cells, and apoptosis of AML cell types, whereas unmodified PU-H71 had no effect. In terms of molecular recognition, mitochondrial targeting of PU-H71 resulted in greater selectivity for mitochondrial *vs*. cytosolic Hsp90, concurrently with a 10-fold lower binding energy with the mitochondrial Hsp90 homolog, TRAP1. Finally, bioinformatics studies identified AML as a unique tumor type potentially “addicted” to mitochondrial Hsp90s, opening fresh therapeutic opportunities in these patients.

Despite evidence that Hsp90-directed protein homeostasis, or proteostasis contributes to tumorigenesis [20], the therapeutic targeting of this pathway has been largely disappointing in the clinic. Although chemically diverse, potent inhibitors of Hsp90 ATPase activity have been developed, these compounds showed modest, if at all, clinical activity, hampered by off-target effects and significant toxicity [21, 26]. The data presented here demonstrate that at least two of these agents with a geldanamycin (17-AAG)- or purine-based chemical scaffold fail to accumulate in mitochondria, leaving unscathed the tumor-promoting activities [2, 3] provided by mitochondrial Hsp90s [13]. Whether this explains their minimal activity in the clinic remains to be fully elucidated. However, data presented here demonstrated that mitochondrial targeting of PU-H71 [25] was sufficient to convert its limited pro-apoptotic activity against epithelial or AML tumor models into effective tumor cell killing through a novel, “mitochondriotoxic” mechanism of action of simultaneous inhibition of bioenergetics and induction of apoptosis.

Overall, our results agree with the findings of an independent study, where PU-H71 was also conjugated to TPP to enable mitochondrial targeting [27]. However, important differences were also observed. In our hands, PU-H71-TPP proved less effective than Gamitrinib at inhibition of oxidative phosphorylation and induction of apoptosis. In addition, our ligand-binding simulation studies suggested that PU-H71 preferentially docked with mitochondrial Hsp90, as opposed to TRAP1 [27], whereas, conversely, Gamitrinib was predicted to bind more tightly to TRAP1, compared to Hsp90. Future studies will determine whether these differences reflect non-overlapping functions of mitochondrial Hsp90 *versus* TRAP1 in enabling mitochondrial reprogramming in cancer.

Despite mechanistic evidence that mitochondrial Hsp90s preserve organelle bioenergetics and cell survival in cancer [16, 17], and that this pathway is important for tumor progression in patients [28], the precise role of these molecules in oxidative metabolism and tumor growth has been debated [22]. Here, the cellular response induced by H71-TPP-2 confirmed the specificity of earlier findings with Gamitrinib, and reinforced a key requirement of mitochondrial Hsp90s for tumor maintenance [13]. Genetic evidence *in vivo* further supports this conclusion, as homozygous deletion [22] or, conversely, transgenic expression [19] of TRAP1 is associated with either delayed or accelerated tumorigenesis, respectively. Taken together, these findings reaffirm a pivotal role of mitochondrial Hsp90s in metabolic and anti-apoptotic reprogramming in cancer, maintaining oxidative bioenergetics and heightened cell survival via increased organelle protein folding [15, 16].

Although TRAP1 is overexpressed in disparate cancers, compared to normal tissues [14], bioinformatics studies reported here identified AML as a tumor type uniquely “addicted” to mitochondrial Hsp90s. This is in line with other data that AML relies on mitochondrial biogenesis [29] and protein translation [30] for disease maintenance, whereas targeting oxidative phosphorylation may overcome treatment resistance [31]. Consistent with this view, Gamitrinib and PU-H71-TPP delivered potent anti-leukemic activity as monotherapy or in combination with Ara-C in AML models, including patient-derived samples, with minimal toxicity for normal PBMC.

In sum, we have shown that mitochondrial targeting of small molecule Hsp90 inhibitors can be successfully achieved across different chemical scaffolds [21], creating more potent anticancer agents compared to the untargeted compounds, characterized by a new, “mitochondriotoxic” mechanism of action. These data reinforce the role of mitochondrial functions as critical tumor drivers [13], and actionable therapeutic targets for molecularly and genetically heterogeneous malignancies, including AML.

## MATERIALS AND METHODS

### Chemical synthesis

All commercial reagents were used as received without further purification. Commercially available anhydrous grade solvents were used. The chemical synthesis and preclinical characterization of first-in-class, mitochondria-targeted small molecule Hsp90 inhibitor, Gamitrinib has been reported [23]. A small molecule mitochondrial oxidative phosphorylation Complex II inhibitor, thenoylfluoroacetone (TTFA) was used as described [17]. Unless otherwise stated, reactions were run under a dry nitrogen atmosphere. Reaction progress was monitored by LC-MS analysis using an Agilent Technologies 1200 Series LC system (Phenomenex 5u Gemini C-18 column) coupled to a 6130 Quadrupole MS. Chromatographic separation was performed using a Gilson 281 automated HPLC system using a Phenomenex 5u Gemini c-18 column. Purity of all final compounds was determined to be ≥ 95 % by reversed phase HPLC on a C-18 column using a gradient of 10-100% acetonitrile in water with 0.1% formic acid or trifluoroacetic acid as a modifier and UV detection at both 254 nm and 280 nm. Proton nuclear magnetic resonance (^1^H-NMR) spectra were recorded on a 400 MHz Bruker Avance III spectrometer at ambient temperature. 6-Amino-9H-purine-8-thiol and 6-bromohexyltriphenylphosphonium bromide were synthesized according to previously reported procedures [23]. ^1^H-NMR and LC/MS spectroscopic data were in agreement with those previously reported [23].

### Synthesis of 6-(6-amino-8-((benzo[d][1,3]dioxol-5-ylthio)-9H-purin-9-yl)hexyl)triphenylphosphonium bromide formate (H71-TPP-1)

#### 8-(Benzo[d][1,3]dioxol-5-yl)thio)-9H-purin-6-amine

To a dry 50 ml round bottom flask was added 6-amino-9H-purine-8-thiol^1^ (3.80 g, 22.73 mmol), 5-iodobenzo[d][1,3]dioxole (11.27 g, 45.46 mmol, BINAP (2,2’-bis9diphenylphosphino)-1,1’-binaphthyl, 1.42 g, 2.27 mmol), potassium t-butoxide (5.10 g, 45.46 mmol) and anhydrous dimethylformamide (10 ml). The resulting stirred solution was degassed under vacuum and placed under a nitrogen atmosphere and then tris(dibenzylideneacetone)dipalladium(0) (1.18 g, 1.14 mmol) was added. The reaction mixture was degassed under vacuum again, placed under a nitrogen atmosphere and then stirred for 18 h at 80°C. The resulting purple reaction mixture was cooled to 22°C, diluted with a mixture of dichloromethane/ethyl acetate/methanol (4:4:1) and filtered through a silica gel pad. The silica pad was washed with the solvent mixture and the combined filtrates were concentrated on a rotary evaporator to remove the volatile solvents and then on a Genevac evaporator to remove the remaining dimethylformamide. The resulting oily solid was triturated with chloroform and the desired product was collected by vacuum filtration to give 2.19 g (34% yield) of a tan solid. ^1^H-NMR (DMSO-d6) δ 8.05 (s, 1H), 7.23, (s, 2H), 7.12 (s, 1H), 7.06 (s, 1H), 6.95 (s, 1H), 6.08 (s, 2H); MS (ESI+) *m/z* = 288 (M+H)^+^.

#### 6-Bromohexyltriphenylphosphonium bromide

A mixture of triphenylphosphine (5 g, 19.1 mmol) and 1,6-dibromohexane (23.3 g, 95.5 mmol) was stirred for 5 h at 90°C. The resulting reaction mixture was cooled for 3 h at 22°C, during which time a sticky precipitate formed. The excess 1,6-dibromohexane was decanted off and the resulting sticky solid was stirred with diethyl ether for 3 h, during which time it became a free-flowing solid. The solid was collected by vacuum filtration, washed with diethyl ether and dried in vacuo to give the desired product (8.59 g, 89% yield) as a white solid.

#### 6-(6-Amino-8-(benzo[d][1,3]diolol-5-ylthio)-9H-purin-9-yl)hexyl)triphenylphos-phonium bromide formate (H71-TPP-1)

A solution of 8-(benzo[*d*][1,3]dioxol-5-yl)thio)-*9H*-purin-6-amine (100 mg, 0.35 mmol) and 6-bromohexyltriphenylphosphonium bromide (197 mg, 0.39 mmol) in anhydrous dimethylformamide (5 ml) was treated with dry cesium carbonate (137 mg, 0.42 mmol). The reaction mixture was stirred for 18 h at 80°C under a nitrogen atmosphere and then cooled to 22°C. The dimethylformamide was removed under high vacuum on a rotary evaporator. The residue was taken up into dimethylsulfoxide, filtered and purified by reversed-phase chromatography (C-18 column, gradient of acetonitrile/water with 0.1% formic acid as a modifier) to yield the bromide/formate salt of the desired product (110 mg, 42% yield) as a yellow oil. ^1^H-NMR (DMSO-d6) δ 8.17 (s, 1H), 7.82-7.60 (m, 15H), 6.89 (s, 1H), 6.74 (s, 1H), 6.60 (s, 1H), 6.04 (s, 2H), 5.48 (br s, 2H), 4.22 (t, J = 7.0 Hz, 2H), 3.69 (m, 2H), 1.79 (m, 2H), 1.40-1.24 (m, 6H); MS (ESI+) *m/z* = 632 (M)^+^.

### Synthesis of 6-(6-amino-8-((6-iodobenzo[d][1,3]dioxol-5-yl)thio)-9H-purin-9-yl)hexyl)triphenyl-phosphonium bromide formate (H71-TPP-2)

#### 8-((6-Iodobenzo[d][1,3]dioxol-5-yl)thio)-9H-purin-6-amine

A solution of 8-(benzo[*d*][1,3]dioxol-5-yl)thio)-*9H*-purin-6-amine (600 mg, 2.09 mmol), trifluoroacetic acid (810 μl) and N-iodosuccinimide (2.82 g, 12.54 mmol) in acetonitrile (25 ml) was stirred under a nitrogen atmosphere for 24 h at 22°C. The resulting reaction mixture was cooled to 22°C, neutralized with saturated aqueous sodium bicarbonate and concentrated to dryness on a rotary evaporator. The resulting brown residue was taken up in acetonitrile, filtered through a plug of glass wool, and chromatographed on silica gel (4:4:1 dichloromethane/ethyl acetate/methanol) to give the desired product as a yellow solid (0.56 g, 48% yield). ^1^H-NMR (DMSO-d6) δ 8.17 (s, 1H), 7.39 (s, 1H), 7.12 (s, 1H), 6.03 (s, 2H); MS (ESI+) m/z = 414 (M+H)^+^.

#### 6-(6-Amino-8-((6-iodobenzo[d]-[1,3]dioxol-5-yl)thio)-9H-purin-9-yl)hexyl)-triphenyl-phosphonium bromide formate (H71-TPP-2)

A solution of 8-((6-Iodobenzo[*d*][1,3]dioxol-5-yl)thio)-*9H*-purin-6-amine (45 mg, 0.11 mmol) and 6-bromohexyltriphenylphosphonium bromide (40 mg, 0.16 mmol) in anhydrous dimethylformamide was treated with dry cesium carbonate (43 mg, 0.13 mmol). The resulting reaction mixture was stirred for 18 h at 80°C under a nitrogen atmosphere and then cooled to 22°C. The dimethylformamide was removed under high vacuum on a rotary evaporator. The residue was taken up into dimethylsulfoxide, filtered and purified by reversed-phase chromatography (C-18 column, gradient of acetonitrile/water with 0.1% formic acid as a modifier) to yield the bromide/formate salt of the desired product (30 mg, 31% yield) as a tan oil. ^1^H-NMR (CDCl_3_) δ 8.24 (s, 1H), 7.88-7.65 (m, 15H), 7.29 (s, 1H), 6.92 (s, 1H), 6.02 (s, 2H), 5.58 (br s, 2H), 4.17 (t, J = 7.0 Hz, 2H), 3.65 (m, 2H), 1.70 (m, 2H), 1.34-1.19 (m, 6H); MS (ESI+) *m/z* = 758 (M)^+^.

### Molecular dynamics (MD) simulations

The crystal structure of Hsp90 in complex with ADP (PDB ID: 2IOP [32]), the cryoEM structure of Hsp90 bound to Cdc37 and Cdk4 (PDB ID: 5FWP [33]) and TRAP1 (PDB ID: 4IPE) [34] were downloaded from the RCSB protein data bank repository (www.rcsb.org). The protein preparation wizard of Schrödinger suite [35] was used to add missing hydrogen atoms, amino acid side chains and missing short loops, to adjust atomic bond orders, and to assign hydrogen bond network. The binding pocket was prepared for docking using Glide [36]. The atomic coordinates of the native ligand were considered as the centroid of the docking box. The polar hydrogen atoms of the amino acids were allowed to rotate during optimization of the docking poses. The binding modes of the ligand in the two proteins were predicted by Glide SP method. To allow for certain protein flexibility, the van der Waals radii were scaled by a factor of 0.8 to soften the potential of the nonpolar regions of the binding pocket. The binding free energy (ΔG_Bind_) of the docked poses was calculated using Prime MM-GBSA method [37]. ΔG_Bind_= ΔE_MM_ + ΔG_SOL_ + ΔGSA, where ΔE_MM_ is the difference in the Prime optimized energies between the complex and the sum of those of the free ligand and receptor. ΔG_SOLV_ is the GBSA solvation energy difference. ΔG_SA_ is the surface area energies difference.

The preparation and simulations of the predicted binding modes were carried out using Desmond molecular dynamics, version 4.4 [38]. The protein complexes were enclosed by solvated boxes of water molecules (TIP4P model). Short MD simulations for 100 ps were carried out to minimize and relax the systems. NTP ensemble was employed for 20 ns MD simulation.

### LC/MS quantification of subcellular compound accumulation

PC3 cells treated with the various unmodified or mitochondria-targeted Hsp90 inhibitors were subcellularly fractionated in cytosol (about 1 mg/ml protein) or mitochondrial (about 1.7 mg/ml) extracts, as described [14]. Protein concentration in the individual fractions was determined using a micro BCA^TM^ Protein Assay Kit (ThermoFisher Scientific, Waltham, MA) with BSA as a standard. Protein aliquots precipitated from the various samples were diluted 3- to 15-fold with water by adding 3 volumes of acetonitrile containing 0.1 μM triphenylphosphonium (TPP) bromide, followed by centrifugation for 10 min at 2000 *g*. Supernatants were transferred to a 96-well polypropylene plate and sealed with a cap mat for UPLC/MS analysis. Compound concentrations (in triplicate) were determined using a Waters Aquity UPLC/Xevo TQ MS system. Five μl samples were fractionated on a Waters Aquity UPLC BEH C18 1.7 μm column (2.1 × 50 mm) equipped with a Vanguard Aquity UPLC BEH C18 1.7 μM precolumn (2.1 × 5 mm). The column was run at 40°C using a 3 min 5-95% acetonitrile gradient containing 0.1 % formic acid (% acetonitrile: 0-0.3 min, 5%; 0.3-1.3 min, 5-40%; 1.3-1.8 min, 40-95%; 1.8-2.3 min, 95%; 2.3-2.4 min, 95-5%; 2.4-3.0 min, 5%) at 0.75 ml/min. The mass spectrometer operated in electrospray positive mode with tune conditions of capillary voltage 2.46 kV, source temperature of 150°C, desolvation temperature of 550°C and desolvation gas flow of 900 l/h. Multiple reaction monitoring/liquid chromatography compound methods were as follows: [Compound, precursor ion > product ion; cone voltage (V); collision voltage (V), retention time (min)]: Gamitrinib: 891.47>830.31, 80, 46, 1.83; H71-TPP-1: 316.80 (doubly charged)>121.90; 36, 20, 1.67; PU-H71: 288.08>166.00, 66, 22, 1.83. Analytes were quantified with Waters MassLynx v4.1 software (Waters Corporation, Milford, MA) using 11-point standard curves with TPP bromide as an internal standard.

### Cell lines and antibodies

Prostate adenocarcinoma PC3, glioblastoma LN229, and acute myeloid leukemia (AML) HL-60, THP-1, Kasumi-1 (Kas-1), MV-411 cells were obtained from the American Type Culture Collection (ATCC, Manassas, VA) and maintained in culture as recommended by the supplier. The T lymphoblastoid cell line CCRF-CEM and chronic myeloid leukemia K562 were from ATCC. A clone of MV-411 cells stably transfected with luciferase (MV-411 Luc) was used. The following primary, patient-derived AML samples devoid of personal identifiers and obtained by pheresis from the Hospital of the University of Pennsylvania #3965 (89% blasts), #4412 (% blasts not available) and #4943 (91% blasts) were used. The following antibodies to cleaved caspase-3, PARP, TRAP1 and Hsp70 (all from Cell Signaling) were used. An antibody to β-actin was from Sigma-Aldrich.

### Protein analysis

Protein lysates were prepared from the different cell types in RIPA buffer (150 mM NaCl, 1.0% Triton X-100, 0.5% sodium deoxycholate, 0.1% SDS, 50 mM Tris, pH 8.0) containing EDTA-free Protease Inhibitor Cocktail (Roche) and Phosphatase Inhibitor Cocktail 2 and 3 (Sigma-Aldrich). Equal amounts of protein lysates were separated by SDS gel electrophoresis, transferred to PVDF membranes and incubated with primary antibodies of various specificities. Protein bands were detected by chemiluminescence.

### Quantification of cell viability

Various tumor cell types were seeded in triplicate onto 96-well plates at 10×10^3^ cells/well, treated with vehicle, or the various unmodified or mitochondria-targeted Hsp90 inhibitors (0-20 μM) for 18 h, and analyzed for changes in cell viability by fluorogenic alamarBlue® quantification. For analysis of mitochondrial electron transport chain, treated cells were analyzed by a (3-(4,5-dimethylthiazol-2-yl)-2,5-diphenyltetrazolium bromide) MTT assay, as described [23]. To quantify changes in mitochondrial membrane potential, HL-60 cells were labeled with the JC1 dye and analyzed for changes in red/green (FL2/FL1) fluorescence ratio, by multiparametric flow cytometry, as described [23]. For determination of apoptosis, PC3 cells (1×10^6^) were labeled for Annexin V and propidium iodide (PI) (BD Biosciences), and analyzed by multiparametric flow cytometry (BD), with quantification of cells in the various apoptotic stages, as described [23].

### Metabolic assays

The individual tumor cell types were incubated with unmodified or mitochondria-targeted Hsp90 inhibitors for 5 h and changes in lactate production were measured by analysis of lactate-dependent conversion of NADP to NADPH in the presence of excess lactate dehydrogenase (LDH), and quantified by absorbance at 450 nm. All assays were performed at 25°C under conditions of linear lactate-limited NADPH formation [18]. For determination of oxygen consumption rates (OCR), treated tumor cells were analyzed with a fluorescence oxygen sensitive probe-based oxygen measuring kit (Luxcel Bioscience). For these experiments, tumor cells were plated on black body, clear bottom 96-well plates, mixed with the various agents in 150 μl of phenol-free DMEM containing 10% FBS and further incubated with an oxygen-sensing probe (10 pmol/well), as described [17]. After 2 h incubation at 37°C, OCR was determined by quantifying the probe fluorescence signal in each well using a plate reader (Beckman Coulter) with excitation and emission wavelengths at 370 nm and 625 nm, respectively [18]. Intracellular ATP content was determined using a luciferin-luciferase method (Biochain) using a microplate luminometer (Beckman Coulter) against standard ATP solutions used as reference, as described [18].

### Bioinformatics analysis

TCGA data set level 3 RNA-seq v2 gene expression and clinical information for LAML cancer were downloaded from the Firehose repository (http://gdac.broadinstitute.org) and log2 transformed. Spearman correlation for each gene *versus* expression of TRAP1 was calculated and results with FDR<5% (estimated by Storey et al [39]) and absolute correlation coefficient of at least 0.3 were considered significant. The average expression of the genes significantly correlated with TRAP1 was calculated, and tested for association with overall survival using Cox regression analysis with age and gender included as additional factors. A Kaplan-Meier curve was plotted by separating patients into two groups based on the median of the average expression profile. TRAP1 correlated genes were then annotated with mitochondrial-related gene ontology cellular components (CC) and biological processes (BP), and overlapped with mitochondrial proteins found to require TRAP1 for proper folding and activity [17]. Significance of overlaps with mitochondrial annotations and protein study was estimated by hypergeometric test. Directional connections between picked mitochondrial-related GO BP and CC categories were then calculated for all A-B pairs as a percent of genes from category A shared with category B. The categories, their connections, and number of TRAP1 associated on mRNA or protein level genes were then visualized in a model.

### Statistical analysis

Data were analyzed using the two-sided unpaired *t* tests using a GraphPad software package (Prism 6.0) for Windows. For all experiments, data are expressed as mean±SD of individual replicates of representative experiments out of at least two or three independent determinations. A *p* value of <0.05 was considered as statistically significant.

### Data availability

The datasets generated and analyzed during the current study are available from the corresponding author on reasonable request.
